# Clinical Studies on the Origin of Leukocytolysins and Antileukocytolysins

**Published:** 1913-10

**Authors:** 


CLINICAL STUDIES ON THE ORIGIN OF LEUKOCYTOLYSINS AND ANTILEUKOCYTOLYSINS, I. I. Manoukhine, (*Arch. des Mal du Coeur, des Vaisseaux, et due Sang,* 1913, VI, 81.) The author has shown in previous papers that the blood contains leukocytolysins and antileukocytolysins, and that organism employs these ferments to increase or to diminish the number of leukocytes according to its necessities in the struggle against infectious agents. The leukocytolytic function of the spleen is demonstrable in the treatment of leukemia by X-rays. In a case of myeloid leukemia under the care of the author the patient's serum was capable of destroying from 16 to 44 per cent. of the leukocytes in the shed blood. The author says that the X-rays in weak therapeutic doses stimulate the activity of the tissue cells. In larger doses the X-rays produce more or less profound changes in the cells. But whether radiation stimulates the activity of an organ or provokes the destruction of its cellular elements, it is evident that any increase of substances after the radiation is due only to the activity of the cells of the radiated organ. In a case of myeloid leukemia the serum of the patient was more pronouncedly leukocytolytic after radiation than before radiation. In the normal individual the leukocytolytic power of the blood was increased after exposing the spleen to the action of the X-rays. When, on the other hand, the thyroid body of a case of Basedow's disease was treated with X-rays the leukocytolytic power of the patient's serum was not increased. The author is of the opinion that the spleen is the organ in which leukocytolysins are produced, and that antileukocytolysins are manufactured in the liver. He suggests that perhaps the increase in the size of the liver and the spleen in cases of infectious disease is due to the attempt to regulate the leukocytolytic property of the blood. He has some reason to think that the liver arrests particularly the destruction of the polymorphonuclear leukocytes, whilst the thyroid body favors the increase of the uninuclear cells in the blood.

